# Large Evolutionary Rate Heterogeneity among and within HIV-1 Subtypes and CRFs

**DOI:** 10.3390/v13091689

**Published:** 2021-08-26

**Authors:** Arshan Nasir, Mira Dimitrijevic, Ethan Romero-Severson, Thomas Leitner

**Affiliations:** Theoretical Biology and Biophysics, Los Alamos National Laboratory, Los Alamos, NM 87545, USA; anasir@lanl.gov (A.N.); mira@lanl.gov (M.D.); eoromero@lanl.gov (E.R.-S.)

**Keywords:** HIV-1, phylogenetics, evolutionary rate, subtypes

## Abstract

HIV-1 is a fast-evolving, genetically diverse virus presently classified into several groups and subtypes. The virus evolves rapidly because of an error-prone polymerase, high rates of recombination, and selection in response to the host immune system and clinical management of the infection. The rate of evolution is also influenced by the rate of virus spread in a population and nature of the outbreak, among other factors. HIV-1 evolution is thus driven by a range of complex genetic, social, and epidemiological factors that complicates disease management and prevention. Here, we quantify the evolutionary (substitution) rate heterogeneity among major HIV-1 subtypes and recombinants by analyzing the largest collection of HIV-1 genetic data spanning the widest possible geographical (100 countries) and temporal (1981–2019) spread. We show that HIV-1 substitution rates vary substantially, sometimes by several folds, both across the virus genome and between major subtypes and recombinants, but also within a subtype. Across subtypes, rates ranged 3.5-fold from 1.34 × 10^−3^ to 4.72 × 10^−3^ in *env* and 2.3-fold from 0.95 × 10^−3^ to 2.18 × 10^−3^ substitutions site^−1^ year^−1^ in *pol*. Within the subtype, 3-fold rate variation was observed in *env* in different human populations. It is possible that HIV-1 lineages in different parts of the world are operating under different selection pressures leading to substantial rate heterogeneity within and between subtypes. We further highlight how such rate heterogeneity can complicate HIV-1 phylodynamic studies, specifically, inferences on epidemiological linkage of transmission clusters based on genetic distance or phylogenetic data, and can mislead estimates about the timing of HIV-1 lineages.

## 1. Introduction

HIV-1 is one of the deadliest pathogens known to mankind. Since the 1980′s, the virus has claimed >30 million lives and infected >75 millions worldwide [1]. The introduction of antiretroviral therapy (ART) around 1995 significantly lowered the HIV-1 mortality rate (from 1.95 million deaths in 2006 to 0.95 million in 2017) [2]. However, HIV prevalence has continued to rise steadily, especially in Sub-Saharan Africa (SSA) [3]. The development of an effective global vaccine is ongoing [4], but is challenged by the rapid evolution and recombination of HIV resulting from a combination of genetic, social, and epidemiological factors.

For example, the HIV-1 reverse transcriptase enzyme lacks proof reading ability and generates significant genetic diversity due to mutations during essentially every round of replication [5]. These mutations are then subjected to different evolutionary pressures exerted by the host immune system and clinical management, which select for immune and drug-escape variants [6,7]. In turn, recombination can not only produce chimeric lineages from existing lineages that have evolved within a single infected person, but also among different subtypes in co-infected persons. These recombinants can cause their own outbreaks, and rise to high prevalence, as seen in several countries [8]. At the population level, natural selection favors strains that are more transmissible between hosts [9]. This selection pressure can offset some of the within-host evolution that is geared towards adapting to a single host and results in differences in the estimates of evolutionary rates between within- and between-host levels [9,10,11]. In addition, the rate of epidemic spread is inversely correlated to virus evolution and faster epidemics, such as in people who inject drugs, typically transmitting highly-similar viruses and, therefore, a slower rate of evolution [12,13]. Thus, HIV-1 evolution is driven by a range of factors involving virus replication enzymes, human immune response, access and cost of ART, social behavior, and population demographics, resulting in an immense genetic diversity among HIV-1 sequences worldwide.

In this study, we calculated the evolutionary (substitution) rates for major subtypes and circulating recombinant forms (CRFs) of the HIV-1 M group, which is responsible for the major AIDS pandemic. In theory, the evolutionary rate should encompass the rate of mutations, substitutions (mutations that become fixed in a population), insertions and deletions (indels), recombination, inversions, and duplications, among other evolutionary events. However, calculation of all these processes is not straightforward, though some success has been achieved in estimating indel rate variation in the hypervariable *env* loop regions [14]. Therefore, while we use the terms evolutionary and substitution rate interchangeably throughout this manuscript, in reality, the analysis describes substitution rate heterogeneity in the HIV-1 M group. To accomplish this task, we performed a systematic analysis of the *pol* and *env* genetic regions that are subject to ART and host immune response selection pressures, and thus have clinical and historical significance. We aimed to include a large number of subtypes/CRFs in our analysis and collected maximum genetic data covering the widest possible temporal (1981–2019) and geographical (100 countries) range. We uncovered significant rate heterogeneity both between and within subtypes that has implications for the phylogenetic and phylodynamic studies of HIV-1. For example, not accounting for rate heterogeneity may mislead inferences of epidemiological events in phylodynamic analyses and can bias the estimates about the origin of major HIV-1 lineages [15]

## 2. Materials and Methods

### 2.1. Data Retrieval

We downloaded a total of 165 *env* and 207 *pol* datasets from the Los Alamos HIV database (27 August 2020) [16]. These datasets included *pol* and *env* genetic sequences from all known subtypes, CRFs, and unique recombinant forms (URFs) of the HIV1-M group. All sequences that included the *env* HXB2 coordinates (6813–7376, C2-V3-C3 region, 564 bp) and *pol* HXB2 coordinates (2253–3308, 1056 bp) were included. These coordinates were chosen to ensure both a broader sampling across subtypes and CRFs (Figures S1 and S2) and keeping in mind their clinical and historical significance. Moreover, the choice of the C2-V3-C3 *env* region was partly inspired by a recent analysis by Palmer and Poon (2019) who calculated indel rates in *env* across major HIV-1 subtypes and CRFs [14], and partly by this region’s demonstrated epidemiological accuracy and popularity in past phylogenetic studies [17]. The HIV-1 M surface envelope glycoprotein (gp120) is notorious for accumulating indels, especially in the regions around the so-called variable loops (V1–V5). Palmer and Poon (2019) showed that V3 accumulates the least and shortest indels, relative to other variable regions [14]. The V3 mediates HIV entry by binding to the host co-receptors. There is therefore stronger selection pressure on V3 to maintain its overall structure and it is thus better suited for inferring substitution rate heterogeneity relative to other *env* variable regions. For all downloaded sequences, we also retrieved metadata about the geo-region (country) of sampling, risk factor, sampling date, HIV treatment status, patient ID, and if the sequence was a member of a previously established epidemic cluster (outbreak).

### 2.2. Data Filtering and Quality Control

Sequence records without sampling dates or coded as problematic (i.e., hypermutants, unique recombinants, or synthetic sequences) were excluded. Moreover, only a single sequence from those appearing in multiple publications was kept. To ensure that we captured the desired regions of interest, all records were pairwise aligned with the HXB2 reference genome and trimmed at the specified coordinates to generate partial *pol* and *env* records. Records that contained >10% gaps were removed. These steps provided us with a total of 78,290 partial *pol* and *env* sequences sampled from 100 countries and covering nearly four decades of sequencing effort (1981–2019). The LANL HIV database included 110 sequences of subtype A, in addition to A1 and A2. Subtype A sequences were therefore re-subtyped using the online COMET HIV-1 tool [18] and pooled with either A1 or A2 depending upon the assignment. Subtype B was the largest dataset in our analysis (26,566 *pol* and 16,271 *env* sequences). For ease of computation, and to minimize any possible confounding or population structure effects from B genetic variants circulating in Asia (e.g., Thai-B or Korean-B) [19], we initially restricted the analysis to B sequences from non-Asian countries, primarily from North America and Europe. Next, we shortlisted seven subtypes and two CRFs that had both *pol* and *env* genetic data from at least 10 different years. These included subtypes A1, A6, B, C, D, F1, and G, as well as CRFs 01_AE and 02_AG that collectively encoded a total of 58,903 sequences (i.e., 75% of the original data). For these datasets, we further removed duplicate/identical sequences (keeping only the earliest record) and kept only one sequence record per patient per year, when the patient ID was known. Similarly, only the earliest sequence from each labeled epidemic cluster was kept.

### 2.3. Dataset Sub-Sampling

Different biological, epidemiological, and social factors can contribute to evolutionary rate heterogeneity among HIV-1 subtypes and CRFs. To fully capture such variability, we reconstructed 100 time-scaled phylogenetic trees for each subtype and CRF, each tree containing no more than randomly sampled 100 taxa. First, we randomly sampled no more than 50 sequences from each year for each subtype/CRF. This step reduced the size of some extremely large datasets corresponding to highly-prevalent or well-studied subtypes such as B and C. Second, we randomly drew no more than 100 sequences from each subtype/CRF, while ensuring all sampling years were represented. In other words, each year contributed at least one sequence. We iterated this approach 100 times to yield 100 datasets for each subtype and CRF and, in total, 900 datasets each for *pol* and *env*, each containing no more than 100 sequences from all sampling years. The sub-sampling strategy provided two major benefits: (i) by restricting to no more than 50 sequences per year, we ensured that we did not overly sample from some of the recent years for which many more sequence records are now available due to the recent revolution in the ease and low cost of genome sequencing, and (ii) by drawing a fixed number of no more than 100 sequences per subtype/CRF, we minimized any biases resulting from incomplete or over-sampling that can disturb the temporal signal in the genetic data for calculation of time-scaled phylogenies [20]. For well-sampled subtypes, there is little chance of a sequence repeating across multiple samples. For subtypes with fewer sequences, however, some sequences can occur in several resamplings making sets not entirely independent from each other. 

### 2.4. Phylogenetic Tree Reconstruction

We aligned the 1800 datasets using MAFFT (version 7.471) [21] (-auto option to determine the optimal alignment strategy depending on the size of the dataset). In parallel, we retrieved the *pol* and *env* alignments for four SIVs (chimpanzee immunodeficiency viruses) from the LANL HIV database to use as outgroups (accession numbers: AF103818, AF382828, AJ271369, and X52154). The *pol* and *env* coordinates for outgroups matched the *pol* and *env* coordinates for HIV-1M datasets. We used the MAFFT–merge function to merge the outgroup and ingroup alignments. The alignments were trimmed to remove sites containing >50% gaps using TRIMAL (version 1.4) [22]. We used IQ-TREE (version 2.0.3) [23] to estimate the maximum likelihood (ML) phylogenetic trees for our datasets. We ran 10 independent tree searches, stopping after 500 unsuccessful iterations in each search, and specified the GTR+G+I substitution model for all searches. We rooted the trees on the branch leading to the SIV outgroup.

### 2.5. Phylogenetic Tree Dating

We used the Least Squares Dating (LSD) program (version 2.0) [24] to estimate the evolutionary rates and dates on the best-scoring ML tree. The LSD program was used as follows: the variance option was set to ‘2’ to run two iterations and to use the branch lengths calculated in the first run to recalculate variances of the branch lengths in the second run. This step is recommended to minimize any effects of potentially long (e.g., outgroup) branches. Furthermore, sequences deemed as outliers (>3 ***Z***-units) by LSD (e.g., weak correlation between sampling dates and phylogenetic placement) and SIV outgroups were removed prior to computing evolutionary rates. Moreover, we set the -u and -l options to 0 to avoid collapsing short branches. This step improves evolutionary rate estimates when the trees contain several short branches. We calculated the 95% confidence intervals (CIs) from 100 samples and specified the lognormal relaxed clock standard deviation to 0.4, previously suggested appropriate for HIV-1 [24]. We set the verbose option to true to also get the root-to-tip (RTT) regression data for all trees and to estimate the relationship between genetic divergence and sampling time for all datasets. Trees where the RTT slope was negative (a total of 39 out of 1800 reconstructions, 2.16%) were excluded from the downstream analysis.

### 2.6. Tip-Date Randomization Test

It is important to verify the existence of a temporal signal in genetic datasets prior to tree dating (i.e., that a positive correlation exists between virus genetic divergence and sampling time). While RTT is a good diagnostic tool to identify problematic sequences and outliers that deviate significantly from a linear model, it is probably (and statistically) inappropriate for phylogenetic data, as all data points have evolved from a common ancestor and hence are dependent [25]. This fact questions the reliability of regression estimates in the standard linear regression models. We therefore re-calculated the substitution rates on the ML trees by randomizing the tip dates within the ingroup. We compared the estimates from random trees versus the actual trees to verify the existence of a temporal signal in genetic datasets.

### 2.7. Rate Autocorrelation Test

We used the CorrTest program [26] to test the null hypothesis that the evolutionary rate is independently distributed across tree branches against the alternative hypothesis that rates are not independently distributed.

### 2.8. Leave-One-Out (LOO) Cross-Validation

We performed LOO cross-validation to evaluate how the subtype/CRF evolutionary rates differed from the global HIV-1M rate. For this purpose, we generated 100 LOO datasets for each subtype and CRF. By definition, the LOO datasets included genetic sequences from all subtypes and CRFs, except the subtype/CRF of interest. Next, we generated time-scaled phylogenies for each LOO dataset and calculated a mean evolutionary rate for each tree. A total of 1800 LOO trees were reconstructed, out of which 1766 (98.1%) passed the RTT and other quality-control tests, described above. Since the LOO datasets included significantly more data (i.e., up to 800 tips in each tree), we performed only a single IQ-TREE search, stopping after 100 unsuccessful iterations. Other settings were kept unchanged. Next, we used the R package *overlapping* (version 1.6) [27] to estimate the overlapping percentage between the estimated density distributions for the evolutionary rates for subtype/CRF of interest and their corresponding LOO trees.

### 2.9. Exploration of Population Structure in Time-Scaled Phylogenies

We used the R package *treestructure* (version 0.1.1) [28] to reveal the underlying population structure in subtype B time-scaled phylogeny. The program was run at 0.005 significance level using a minimum clade size threshold of 50 tips. Treestructure rank-sum test was used to evaluate if the two partitions had evolved under the same coalescent process.

## 3. Results

### 3.1. Substantial Substitution Rate Heterogeneity between HIV-1 Subtypes and CRFs

**Figure 1:**
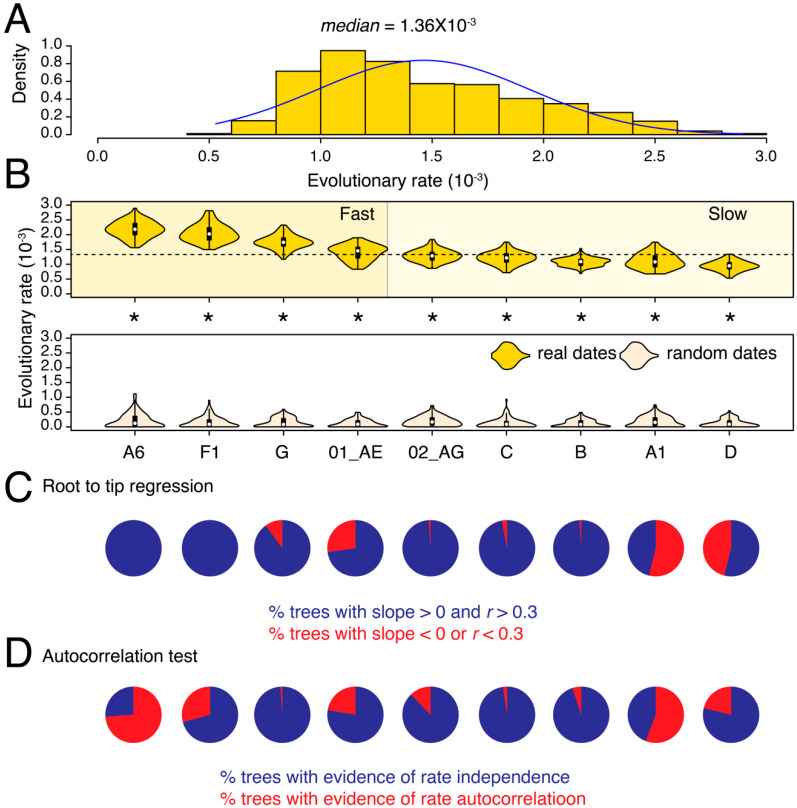
Substitution rate heterogeneity in *pol*. (**A**) The histogram displays the distribution of calculated substitution rates for all 900 *pol* tree reconstructions. Median of the distribution is listed on the top. (**B**) The violin plots show the distribution of substitution rates calculated individually for each subtype/CRF (i.e., across 100 tree reconstructions). The white circle indicates the group median. The dotted horizontal line represents the global median rate calculated in (**A**). Distributions of substitution rates calculated for trees with real and random dates can be compared. All comparisons were statistically significant (indicated by asterisk). (**C**) Pie-charts show the proportion of phylogenetic trees that failed the RTT tests described in text. (**D**) Pie-charts show the proportion of phylogenetic trees with evidence of autocorrelated rates. Note that the latest version of the CorrTest program [26] only works with standard ML trees and not dated trees. The mean and median substitution rates, along with 95% CIs, for each subtype and CRF are also reported in Table 2. Note that Table 2 excludes trees where RTT slope was either negative or *r* was <0.3.

**Figure 2:**
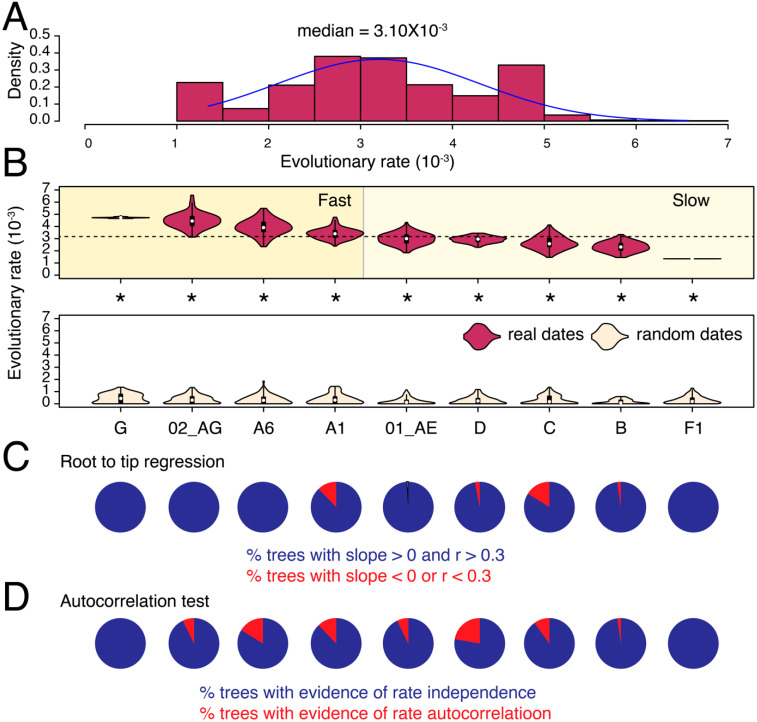
Substitution rate heterogeneity in *env*. The histogram displays the distribution of calculated substitution rates for all 900 *env* tree reconstructions. Median of the distribution is listed on the top. (**B**) The violin plots show the distribution of substitution rates calculated individually for each subtype/CRF (i.e., across 100 tree reconstructions). The white circle indicates the group median. The dotted horizontal line represents the global median rate calculated in (**A**). Distributions of substitution rates calculated for trees with real and random dates can be compared. All comparisons were statistically significant (indicated by asterisk). (**C**) Pie-charts show the proportion of phylogenetic trees that failed the RTT tests described in text. (**D**) Pie-charts show the proportion of phylogenetic trees with evidence of autocorrelated rates. The mean and median substitution rates, along with 95% CIs, for each subtype and CRF are also reported in Table 2. Note that Table 2 excludes trees where RTT slope was either negative or *r* was <0.3.

We calculated the evolutionary (substitution) rates in partial *pol* and *env* genes for nine major HIV-1 M-group subtypes and CRFs that qualified our filtering criteria (see Methods). The median substitution rate for *pol* across all subtypes/CRFs and tree reconstructions was 1.36 × 10^−3^ substitutions site^−1^ year^−1^ (*ssy*) (Figure 1A) compared to 3.10 × 10^−3^ *ssy* for *env* (Figure 2A), confirming faster evolution of *env*, which is well-known to be under diversifying selection in response to host immune surveillance. For *pol*, the median substitution rates differed by 2.3-fold among subtypes and CRFs, from 0.95 × 10^−3^ *ssy* in subtype D to 2.18 × 10^−3^ *ssy* in subtype A6 (*p* < 0.001, two-tailed Mann–Whitney test) (Figure 1B). The difference was even more profound in *env*, where median rates ranged from 1.34 × 10^−3^ *ssy* in subtype F1 to 4.72 × 10^−3^ *ssy* in subtype G, indicating a roughly 3.5-fold difference (*p* < 0.001, two-tailed Mann–Whitney test) (Figure 2B).

In both genes, we could initially classify subtypes as either fast or slow-evolving relative to the global medians (see dotted horizontal lines in Figure 1B and Figure 2B). For example, in *pol*, we classified subtypes A6, F1, G, and CRF 01_AE as fast-evolving, and the rest as slow-evolving (Figure 1B). To validate these classifications, we performed LOO cross-validation and calculated an overlap between the estimated density distributions of the calculated evolutionary rates for each subtype/CRF versus their corresponding HIV-1M (LOO) trees (Figures S3 and S4). The LOO cross-validation revealed that except subtype G and CRF 01_AE, all other subtypes and CRFs indicated a less than 20% overlap in the estimated *pol* evolutionary rates for subtype/CRF versus HIV-1M (LOO) analyses (Figure S3). For example, we noticed a 0% overlap between the slowest-evolving subtype D and HIV-M and only an 8% overlap between the fastest-evolving sub-subtype A6 and HIV-1M (Figure S3). Similarly, in *env*, we initially classified G, 02_AG, A6, and A1 as fast-evolving, and the rest as slow-evolving (Figure 2B). The LOO cross-validation confirmed that the overlap for the fast-evolving 02_AG, A6, and A1 was less than 20% relative to HIV-1M (Figure S4). However, the overlap ranged from 26–77% for the rest, indicating that these rates did not, on average, deviate significantly from the HIV-1M rates. Overall, the LOO cross-validation confirmed the existence of subtype specific evolutionary rates in both *pol* and *env*, especially for the fast-evolving subtypes and CRFs. Taken together, subtype A6 was classified as fast-evolving in both *pol* and *env* analyses (Figure 1 and Figure 2) and indicated very little (8–11%) overlap compared to LOO trees (Figures S3 and S4). Interestingly, subtype B was classified as slow-evolving in both Figure 1 and Figure 2 and had only 2% overlap with LOO trees in *pol* (Figure S3) but a 26% overlap with LOO trees in *env* (Figure S4).

The tip-date randomization test verified the existence of a strong temporal signal in the original trees for all comparisons (*p* < 0.05, two-tailed Wilcoxon signed-rank test for paired samples). Moreover, except A1 in *pol*, >50% of trees in all datasets, passed the RTT test (Figure 1C and Figure 2C, Table 1). Similarly, other than A1 and A6 in *pol*, none of the remaining datasets indicated evidence for autocorrelated rates (Figure 1D and Figure 2D). The calculated rates are therefore well-supported by multiple diagnostic measures (see Table 2 for substitutions rates after removing trees with poor RTT signal). Overall, the analysis revealed substantial substitution rate variation both across different genes in the HIV-1 genome and also among subtypes, which corroborates previous findings [15,29], and indicates that HIV-1M strains are probably evolving under different selection pressures in different geographical and genetic regions.

**Table 1:** Evidence of temporal signal and autocorrelated rates on phylogenetic trees. The values in the table show how many of the 100 trees for each subtype/CRF failed the RTT test (i.e., either negative slope or *r* < 0.3) or indicated evidence of autocorrelated rates, tested via the CorrTest program [26]. A6 and A1 are bolded, as >50% of these trees indicated either evidence of rate autocorrelation or failed RTT tests. See Table S1 for complete data.

	CorrTest		Root-to-Tip Regression	
Subtype/CRF	Autocorrelated	Independent	Pass	Fail
*env*				
01_AE	7	93	99	1
02_AG	7	93	100	0
A1	12	88	88	12
A6	16	84	100	0
B	2	98	98	2
C	10	90	84	16
D	22	78	97	3
F1	0	100	100	0
G	0	100	100	0
*pol*				
01_AE	22	78	73	27
02_AG	12	88	99	1
**A1**	**56**	**44**	**46**	**54**
**A6**	**74**	**26**	**100**	**0**
B	5	95	99	1
C	2	98	97	3
D	21	79	54	46
F1	29	71	100	0
G	1	99	90	10

**Table 2:** Substitution rates in *pol* and *env*. The table below reports the calculated *pol* and *env* substitution rates (×10^−3^ *ssy*) for all analyzed subtypes and CRFs. The mean, median, standard error, and the lower and upper bounds of the 95% CIs are reported. The table is sorted by the median rate, in a descending manner. The table excludes trees that indicated either a negative RTT slope or *r* < 0.3 (see text). See Table S1 for complete data.

Subtype/CRF	Gene	Trees	Mean	Median	Std. Error	Lower-Bound	Upper-Bound
G	*env*	100	4.72	4.72	0.0045	4.71	4.73
02_AG	*env*	100	4.48	4.45	0.0623	4.36	4.60
A6	*env*	100	3.94	3.91	0.0647	3.81	4.06
A1	*env*	88	3.41	3.41	0.0461	3.32	3.50
01_AE	*env*	99	2.99	3.02	0.047	2.90	3.09
D	*env*	97	2.93	2.98	0.0261	2.88	2.98
C	*env*	84	2.74	2.64	0.0555	2.63	2.85
B	*env*	98	2.33	2.32	0.0418	2.25	2.41
F1	*env*	100	1.34	1.34	0.00003	1.34	1.34
A6	*pol*	100	2.18	2.18	0.0275	2.12	2.23
F1	*pol*	100	2.05	2.02	0.0294	1.99	2.11
G	*pol*	90	1.78	1.76	0.0225	1.73	1.82
01_AE	*pol*	73	1.48	1.51	0.0238	1.43	1.52
02_AG	*pol*	99	1.29	1.29	0.0200	1.25	1.33
C	*pol*	97	1.22	1.23	0.0206	1.18	1.26
B	*pol*	99	1.08	1.09	0.014	1.05	1.11
A1	*pol*	46	1.25	1.26	0.0338	1.18	1.31
D	*pol*	54	0.98	0.96	0.0200	0.94	1.02

### 3.2. Substantial Substitution Rate Heterogeneity between Closely-Related HIV-1 Sub-Subtypes

While there were significant rate differences between subtypes, there were also substantial rate differences within subtypes. For instance, sub-subtypes A1 and A6 indicated a significant rate difference in *pol* (2.18 × 10^−3^ vs. 1.08 × 10^−3^ *ssy*; *p* < 0.001, two-tailed Mann–Whitney test). Since 54% of the A1 *pol* trees had failed the RTT test (Figure 1C, Table 1), we reasoned the difference in the evolutionary rates between these closely related sub-subtypes could have arisen from differences in the strength of temporal signal provided by each dataset. To investigate this possibility, we recalculated the *pol* substitution rates for A6 and A1 sub-subtypes by reconstructing new time-scaled phylogenies using larger datasets, including all qualifying sequences up to 50 taxa per year in the rate estimation. We analyzed a total of 426 A6 sequences sampled between 1997–2016 from countries in the Former Soviet Union (FSU) and 244 A1 sequences (1985–2015) from SSA, Europe, Asia, and Oceania.

**Figure 3:**
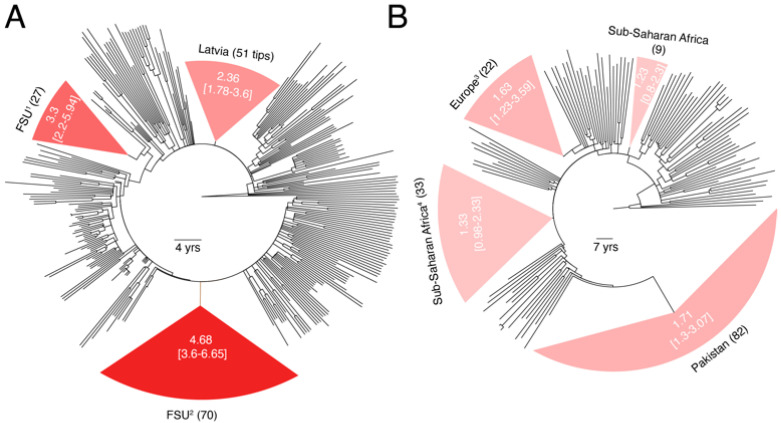
Comparison of *pol* substitution rates between sub-subtypes A6 and A1. (**A**) Dated phylogeny comprising of a larger sampling of A6 sequences is displayed. Partitions corresponding to monophyletic clades of viruses sampled from countries in the FSU are highlighted. Numbers in parentheses indicate total number of tips in a clade. Values inside the clade indicate the calculated mean substitution rate along with the 95% CI for that clade. Rates for the entire tree (i.e., without partitions) and remaining branches (i.e., excluding partitions) were 3.23 × 10^−3^ and 2.98 × 10^−3^, respectively. RTT slope was 4.91 × 10^−3^ (*R*^2^ = 0.477, *r* = 0.69). (**B**) Dated phylogeny comprising of a larger sampling of A1 sequences is displayed. Rates for the entire tree (i.e., without partitions) and remaining branches (i.e., excluding partitions) were 1.62 × 10^−3^ and 1.29 × 10^−3^, respectively. RTT slope was 2.35 × 10^−3^ (*R*^2^ = 0.16, *r* = 0.39). Other details similar to (**A**). ^1^ Includes 26 samples from Russia and 1 sample from Armenia, ^2^ includes 64 samples from Russia, 3 from Uzbekistan, 2 from Armenia, and 1 from Latvia, ^3^ also includes 1 sample from Australia, ^4^ also includes 9 samples from India and 1 sample from Cyprus.

The estimated *pol* substitution rates of sub-subtypes A6 and A1 still differed by 2-folds (3.23 × 10^−3^ vs. 1.62 × 10^−3^ *ssy*) (Figure 3A) and were greater than the global *pol* median (1.36 × 10^−3^ *ssy*) and medians calculated for these sub-subtypes previously in Figure 1 (2.186 × 10^−3^ vs. 1.08 × 10^−3^ *ssy*). Furthermore, the RTT test verified the existence of sufficient temporal signal in both trees (*r* = 0.69 for A6 and 0.39 for A1). In A6, we could identify three monophyletic partitions comprising of individuals sampled in Latvia and other parts of the FSU (mainly Russia). While the 95% CIs for the three A6 partitions overlapped with each other (Figure 3A), they excluded the 95% CIs calculated for A1 partitions (Figure 3B). For example, the largest A6 partition corresponding to individuals sampled in the FSU had a mean rate of 4.68 × 10^−3^ *ssy* (95% CI = 3.6–6.65 *ssy*), which excluded both the mean rates and 95% CIs for all four A1 partitions (Figure 3B). These results therefore suggest that A6 is indeed a fast-evolving HIV-1 variant and evolutionary rate heterogeneity exists even between closely related HIV-1M sub-subtypes.

### 3.3. Substantial Substitution Rate Heterogeneity within Subtypes

Subtype B was amongst the slowest evolving subtypes in both *pol* and *env* analyses (Figure 1 and Figure 2). The B dataset included sequences from mostly North America and European countries, with a presumably higher ART access and enrollment rate, certainly at earlier dates. The dataset thus excluded the two major subtype B genetic variants that circulate in several Asian countries (e.g., the Thai-B in China, Laos, Myanmar, and Thailand, and Korean-B in South Korea) [19]. Therefore, we investigated substitution rate heterogeneity within B by calculating the substitution rates for four major known B genetic variants (i.e., the Brazilian-B, Trinidad and Tobago-B, Thai-B, and the Korean-B) [19], in addition to B sequences sampled from rest of the World (primarily from North America, Europe, and Oceania). We used *env* for this analysis, which by virtue of its faster evolution is better suited to resolve genetic diversity among closely related strains. We analyzed a total of 1153 B sequences (1981–2016), by allowing up to 50 sequences from each year, as above.

**Figure 4:**
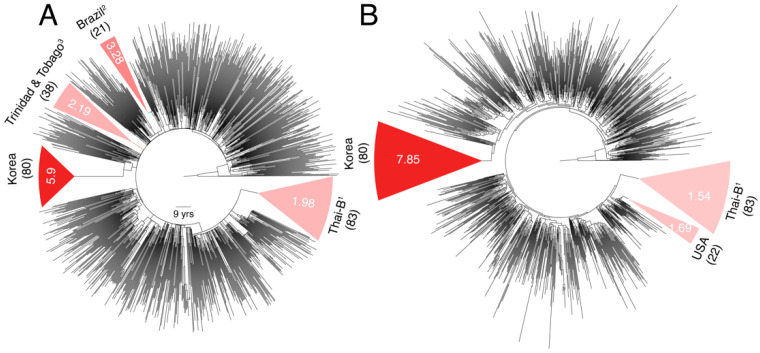
Comparison of *env* substitution rates among subtype B genetic variants. (**A**) Dated phylogeny comprising of a larger sampling of subtype B sequences is displayed. Partitions corresponding to monophyletic clades of B genetic variants sampled in Asia (Thai-B and Korean-B) and South America (Brazilian-B and Trinidad and Tobago B) are highlighted. Remaining branches correspond to B viruses sampled in rest of the World, primarily from North America and Europe. Numbers in parentheses indicate total number of tips in a clade. Values inside the clade indicate the calculated mean substitution rates. The 95% confidence intervals are not shown as they would not be legible (see text). Rates for the entire tree (i.e., without partitions) and remaining branches (i.e., excluding partitions) were 2.90 × 10^−3^ and 2.42 × 10^−3^, respectively. RTT slope was 2.98 × 10^−3^ (*R*^2^ = 0.30, *r* = 0.55). (**B**) The node height of the tMRCA of the Korean-B clade was used to extract monophyletic partitions in the ML tree. Out of the eight identified partitions, three did not have sufficient temporal signal. Two partitions corresponded to the Trinidad and Tobago clade defined in (**A**) but were not analyzed because of the poor RTT signal (negative signal). The rates for the remaining three partitions are displayed. Other details similar to (**A**). ^1^ Includes 72 samples from China, 10 from Thailand, and 1 from Myanmar (1), ^2^ also includes 1 sample from Argentina, 2 from France, 1 from China, 2 from USA, and 1 from Ukraine, ^3^ also includes 1 sample from USA.

The mean substitution rate for the larger subtype B tree was 2.90 × 10^−3^ *ssy* (Figure 4A) compared to 2.31 × 10^−3^ *ssy* calculated previously in Figure 2B, indicating a 25% increase. The mean substitution rates for the four major B genetic variants, in increasing order, were 1.98 × 10^−3^ *ssy* in Thai-B [95% CI = 1.65–2.83 × 10^−3^ ssy], 2.19 × 10^−3^ *ssy* in Trinidad and Tobago (1.61–4.48 × 10^−3^ ssy), 3.28 × 10^−3^ *ssy* in Brazil (2.73–6.23 × 10^−3^ ssy), and 5.9 × 10^−3^ *ssy* in Korea (4.62–7.83 × 10^−3^ ssy). These rates suggested significant rate heterogeneity among subtype B variants, particularly a very fast evolutionary rate in the relatively-younger Korean-B clade (Figure 4A). An earlier study also reported a very high (8.0 × 10^−3^ *ssy*) *env* substitution rate in the Korean-B HIV epidemic but they also reported higher rates for non-Korean B sequences (7.3 × 10^−3^ *ssy*) [30].

Unfortunately, information about the prevalence of ART over time in a population and dates about the origin or introduction of a subtype in a population cannot always be reliably estimated. Hence, we were unable to associate calculated rates to these metadata. Since the Korean HIV epidemic is likely a younger epidemic (with first reported cases in 1985) [31] compared to other subtype B variants, we reasoned that mutation saturation over time could have caused observed sequence divergence to be lower than the actual sequence divergence in the non-Korean B clades. Therefore, we reanalyzed the subtype B tree by extracting virus sequences sampled over similar timescales. We calculated the node height of the tMRCA of the Korean-B clade on the subtype B ML tree, which was used as input to LSD (Figure 4B), and used this value to identify two additional non-Korean monophyletic clades at the same height (Figure 4B). These clades included the entire Thai-B clade (83/83 tips) and another clade of 22 individuals sampled from the USA. We generated new time-scaled phylogenies for both clades, in addition to the Korean clade, and recalculated the evolutionary rates. The Korean clade, once again, indicated a very fast evolutionary rate with a mean of 7.85 × 10^−3^ *ssy* (6.17–10.5 × 10^−3^ *ssy*) versus the Thai-B 1.54 × 10^−3^ *ssy* (0.84–2.51 × 10^−3^ *ssy*) and USA 1.69 × 10^−3^ *ssy* (0.57–3.3 × 10^−3^ *ssy*) clades, thus verifying substantial rate heterogeneity that exists within subtype B and especially a faster genetic evolution of the Korean HIV epidemic.

**Figure 5:**
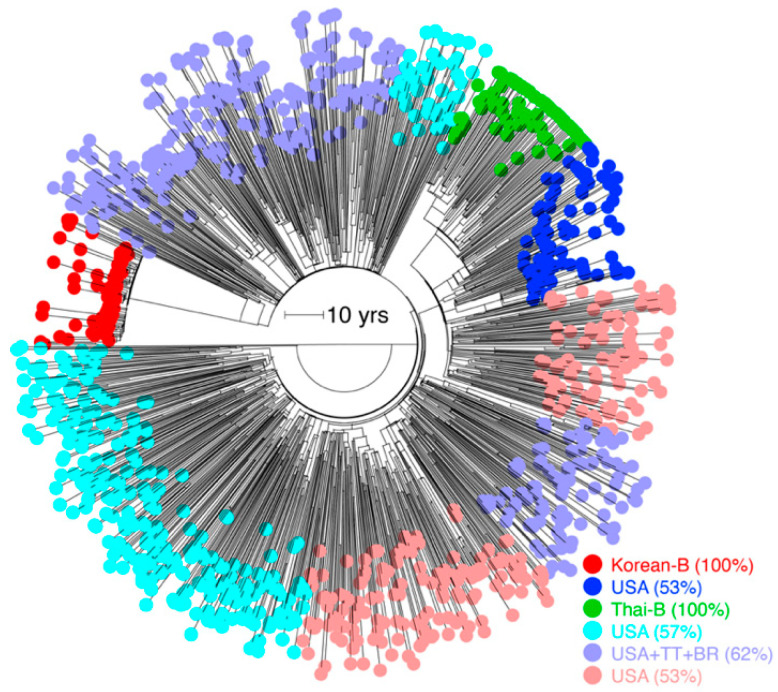
Hidden population structure in time-scaled subtype B tree. Identified partitions are colored individually. Partitions are named by the country or group of countries with >50% sequences in that partition.

The time-scaled subtype B tree suggested the existence of an underlying population structure aligned with geography (Figure 4A). Therefore, we ran the *treestructure* program to reveal six partitions in the time-scaled B phylogeny (Figure 5), shaped either by different epidemiological and demographics histories or the same coalescent processes operating at different degrees [28]. These included a monophyletic partition corresponding to the entire Korean-B clade (80/80 tips, 100%), another monophyletic partition corresponding to the Thai-B clade, and another polyphyletic partition that grouped the Trinidadian and Brazilian-B sequences with the North American and European sequences. The *treestructure* rank-sum test confirmed that each pair of the identified partitions had evolved under a different coalescent process (*p* < 0.05). The analysis thus suggested that the Korean-B had a different epidemiological or demographic history, relative to both the Thai-B and the North American, Trinidadian, and the Brazilian-B clades. For example, the Thai-B epidemic was initially characterized by rapid and explosive spread among injecting drug users in Thailand [19], whereas the Korean epidemic mainly spreads (relatively slowly) via homo- or heterosexual contact [31,32]. The evolutionary rate is thus influenced by the underlying demographic and population history, which is responsible for establishing the virus contact and transmission network and can cause observed rate heterogeneity. The analysis implies that such processes can cause rate heterogeneity even within a subtype.

## 4. Discussion

We performed a systematic calculation of the *pol* and *env* substitution rates in major HIV-1 subtypes and CRFs. Similar to previous analyses [15,29,33], we uncovered significant substitution rate heterogeneity both across the HIV-1 genome, between subtypes, and even within subtypes (i.e., sub-subtypes and genetic variants of the same subtype). Such variability is a likely and natural consequence of the differences in the rates of virus spread in different epidemics and geographics, relative ages of different epidemics unfolding in different parts of the World, and ART availability and enrollment rates, among other virus genetic factors such as fast mutation, replication, and recombination rates.

In general, HIV outbreaks involving rapid virus spread (e.g., among people who inject drugs) typically involve highly-similar viruses and thus report lower evolutionary rates compared to epidemics where virus transmits steadily or slowly in a population [13]. Unfortunately, the metadata about risk route (e.g., sexual vs. intravenous) and gender were largely missing in our filtered datasets. Thus, we could not explicitly analyze such rate heterogeneities. However, the rate of virus spread in a population is not merely a feature of the nature of the outbreak. It is also dictated by the underlying demographic and population structure that may vary geographically. For example, the primary mode of HIV transmission in Korea is via sexual contact (94–98%) [31,32], which explains the three-times slower growth rate observed in the Korean HIV epidemic [30] and contrasts with, for example, the Thai HIV epidemic, which was initially characterized by explosive HIV spread among injecting drug users [19]. We noticed a very fast evolutionary rate in the Korean-B clade compared to the non-Korean B clades, which can be explained by a slower or steadier rise in the number of HIV infections in Korea over time [30].

In addition, several studies have reported a time-dependent bias in the estimation of virus evolutionary rates, with shorter-timescales associated with faster evolutionary rates [34,35]. While several factors have been proposed for such time-dependent bias [36], mutation saturation and purifying selection are perhaps the most influential [35]. For example, mutation saturation can cause the actual genetic diversity to be underestimated and is likely to manifest more profoundly and severely over longer timescales. In turn, purifying selection can remove deleterious mutations over time. However, its effect will likely be limited on sequences sampled over short timescales that may carry several transient and deleterious mutations. In other words, novel genetic variants emerging in new parts of the world are likely to report a faster evolutionary rate until selection has had the time to act. This can be an additional explanation for why we observed faster evolutionary rates in the relatively younger A6 and Korean-B clades compared to A1 and non-Korean B clades, respectively.

Another factor responsible for the observed rate heterogeneity is the access to and cost of ART [12]. ART enrollment can suppress the viral load and decrease the risk of virus transmission [37]. However, access to ART varies significantly across the World, with much higher enrollment rates in North America and Europe compared to rest of the World. Unfortunately, such metadata was largely unavailable for our datasets. For example, the ART enrollment information was missing for the entire Pakistani A1 clade (82 individuals) in Figure 3B. We were therefore unable to ascertain the possible impact of ART enrollment in causing the observed rate heterogeneity. In addition to these biological reasons, technical reasons such as the limitations of phylogeny estimation and tree-dating [20] and biases in the sampling and sequencing trends across the world can also cause differences in the quality of datasets available for such analyses and be ultimately responsible for observed rate heterogeneities. For example, we kept only one sequence from each known outbreak in our analysis. However, the epidemiological connections of the majority of virus sequences are not reported in public databases. Therefore, there is a small risk of including epidemiologically-linked individuals (e.g., monophyletic FSU clusters in Figure 3) that may have biased our rate estimates.

HIV-1M substitution rate heterogeneity can bias our estimates of the ages or dates of evolutionary divergence events. Wertheim et al. (2012) revealed inconsistencies in the estimated ages of HIV-1 subtypes between a combined HIV-1M and subtype-specific phylogenies in the presence of significant rate heterogeneity [15]. They suggested to focus only on single subtype analyses, rather than inferring the tMRCA of all subtypes in a combined HIV-1M phylogeny, as current relaxed clock models may be inadequate for modeling extensive rate heterogeneity among HIV-1 subtypes. We now confirm that rate heterogeneity not only exists between subtypes, but also within subtypes. This was made explicit by drawing 100 random samples 100 times from the same subtype/CRF and recalculating rates on the time-scaled phylogenies (Figure 1 and Figure 2) and also by explicitly quantifying rate heterogeneity among different genetic variants of the same subtype and closely-related sub-subtypes (Figure 3 and Figure 4). Thus, substantial rate heterogeneity, along with the unaccounted factors of mutation saturation and purifying selection, cast doubts on the estimated ages and dates of origins of HIV-1M major lineages.

Not accounting for rate heterogeneity can also misguide epidemiological inferences derived from genetic or phylogenetic data. From a public health perspective, we are interested in separating parts of the trees that are linked by recent, local, and contemporary transmission (e.g., events that happened in the last 10 years) from the rest and presumably epidemiologically-unlinked events. We can use the HIV evolutionary rate to determine the cutoff separating transmission events happened, for example, in the last 10 years from events that happened before. However, the position of the tree cutoff value, a so-called the epidemiological horizon (Nasir et al., manuscript in preparation), will vary based on the input evolutionary rate. Using a single value for all subtypes/CRFs will cause differences in both the numbers and sizes of retrieved transmission clusters. For example, depending on where the 10-year cutoff mark is set, we may risk including longer branches and epidemiologically unlinked individuals. Such problems can even bias tools that do not explicitly rely on phylogenetic trees for inferring transmission clusters such as HIV-TRACE [38]. Depending on how fast or slow the evolutionary rate is, the genetic distance cutoffs used to infer transmission clusters may represent widely different timescales. Such rate heterogeneity should therefore be accounted for when performing HIV-1M phylogenetic and phylodynamic studies and can cause unintended consequences.

## 5. Conclusions

We report substantial evolutionary rate heterogeneity in HIV-1M caused by a combination of genetic, demographic, and technical factors. The numbers reported in this analysis provide a framework for relative comparisons of evolutionary rates among HIV-1M subtypes and CRFs and do not represent absolute differences. The analysis is mostly based on epidemiologically-unlinked individuals (one sequence per patient per year, one member from each known outbreak/cluster). However, the distributions covering the variability in the evolutionary rates for each major subtype/CRF can serve as informative priors for future Bayesian inferences.

## Data Availability

Not applicable.

## Supplementary Materials

The following are available online at https://www.mdpi.com/article/10.3390/v13091689/s1. Figure S1. The distribution of *env* sequences by length in the LANL HIV database. All fragments relate to HXB2 coordinates. Figure S2. The distribution of *pol* sequences by length in the LANL HIV database. All fragments relate to HXB2 coordinates. Figure S3. LOO cross-validation for *pol*. The plots reveal the overlap between the estimated density distributions for each subtype/CRF versus their corresponding LOO datasets. Figure S4. LOO cross-validation for *env*. The plots reveal the overlap between the estimated density distributions for each subtype/CRF versus their corresponding LOO datasets. Table S1. The calculated substitution rates along with 95% confidence intervals, *R*^2^, RTT slope, and *r* values for all tree reconstructions. Trees with a negative slope were removed (reduced 1800 trees into 1761). Trees indicating a weak *r* (i.e., <0.3) between genetic divergence and sampling times are highlighted.
