# Supplementary figures and images for: Large Evolutionary Rate Heterogeneity among and within HIV-1 Subtypes and CRFs
Source: Viruses. 2021 Aug 26;13(9):1689. doi: 10.3390/v13091689 (PMC8473000; doi:10.3390/v13091689)

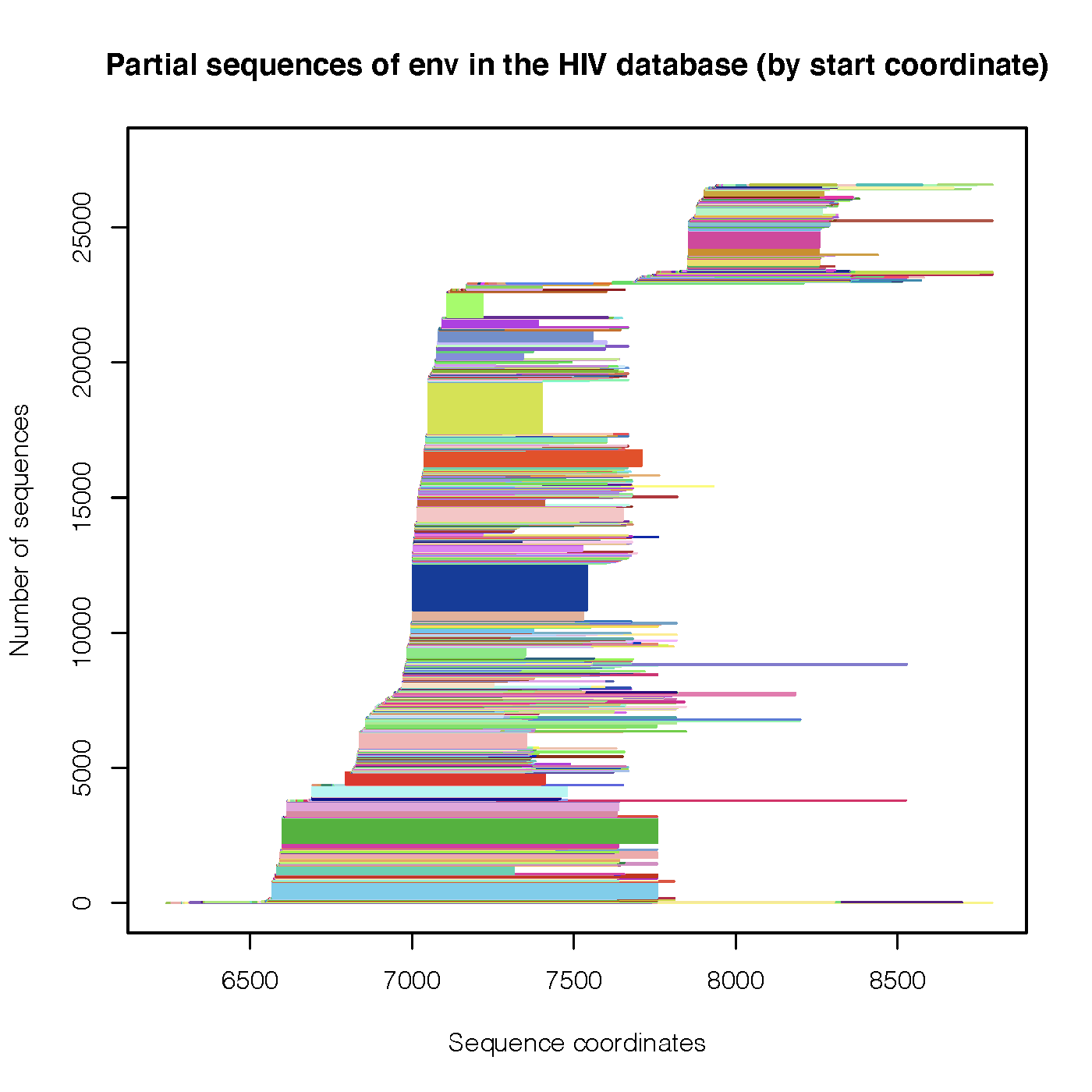

Supplement: Supplementary file 1 [file viruses-13-01689-s001.zip › FigureS1.png]

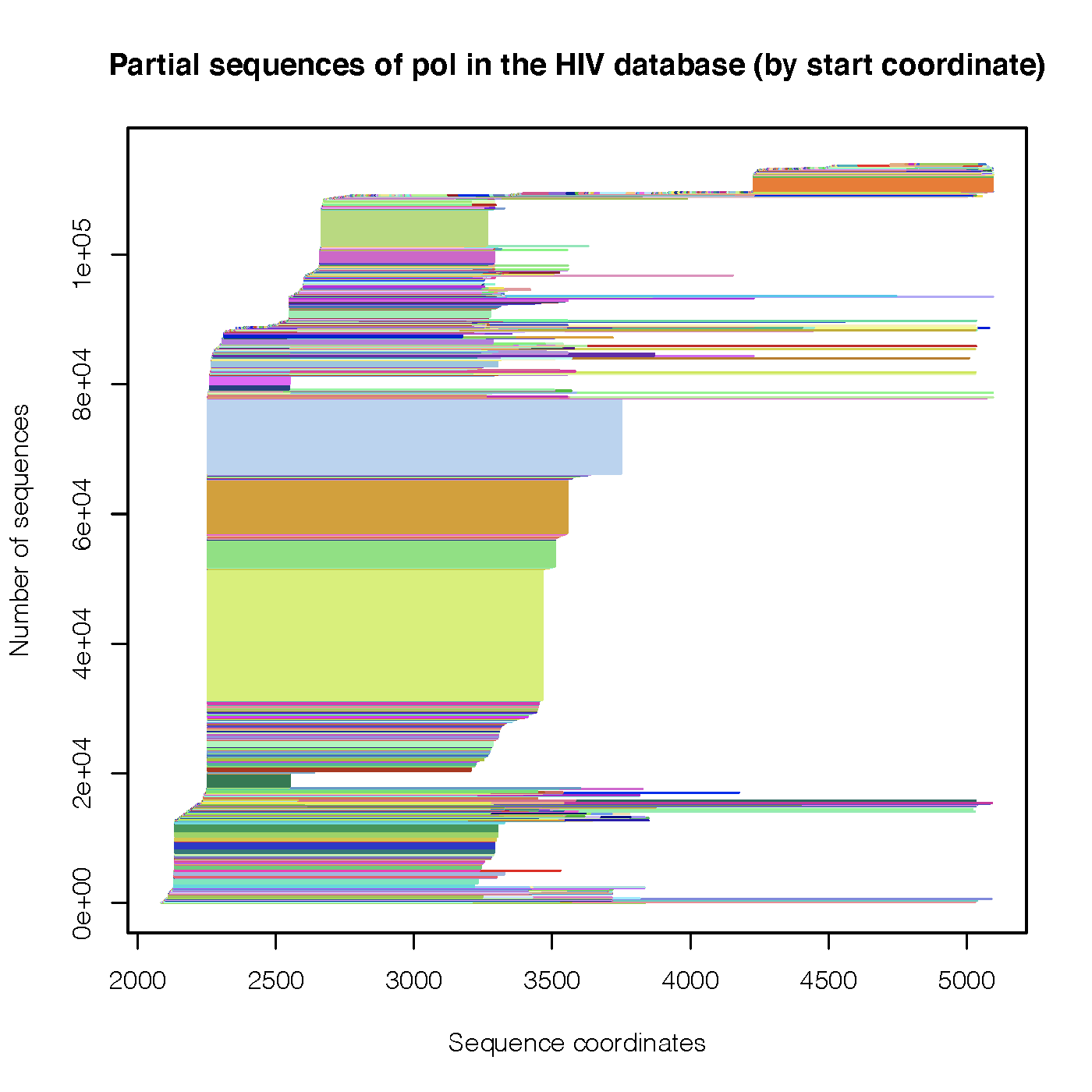

Supplement: Supplementary file 1 [file viruses-13-01689-s001.zip › FigureS2.png]

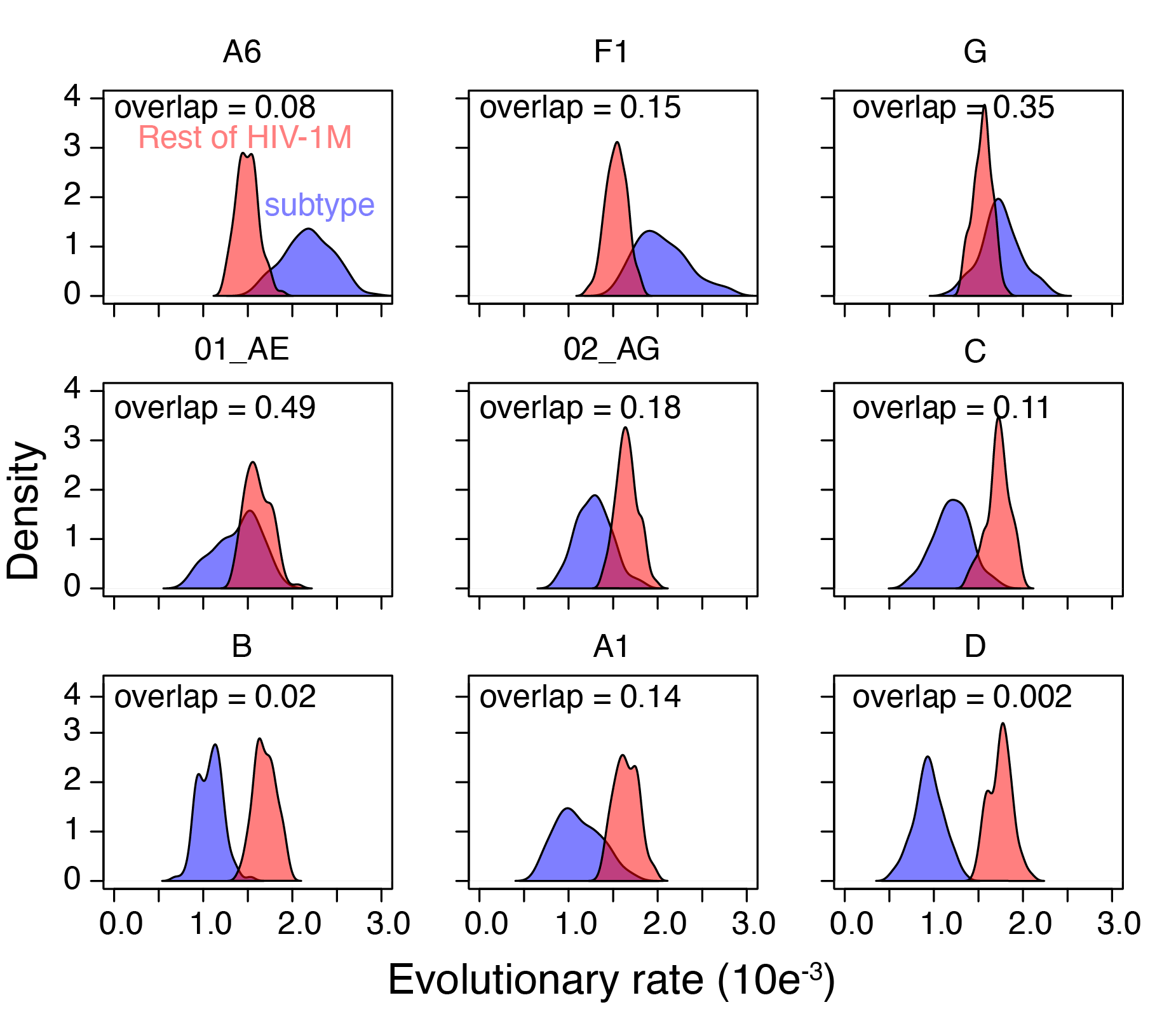

Supplement: Supplementary file 1 [file viruses-13-01689-s001.zip › FigureS3.png]

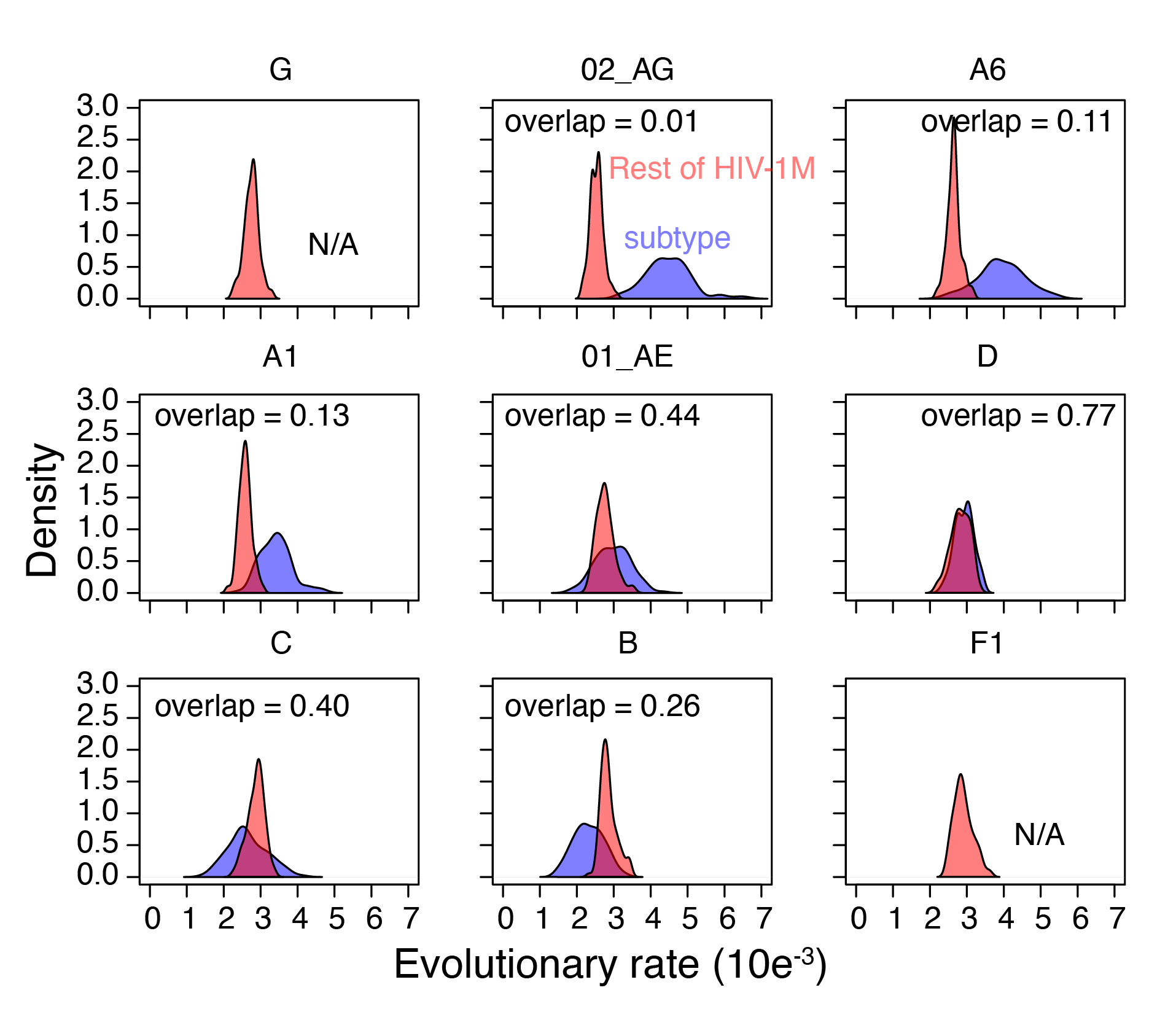

Supplement: Supplementary file 1 [file viruses-13-01689-s001.zip › FigureS4.png]
